# Vascular Protective Effect and Its Possible Mechanism of Action on Selected Active Phytocompounds: A Review

**DOI:** 10.1155/2022/3311228

**Published:** 2022-04-16

**Authors:** Nur Aqilah Kamaruddin, Muhammad Nazrul Hakim Abdullah, Jun Jie Tan, Vuanghao Lim, Lai Yen Fong, Siti Aisyah Abd Ghafar, Yoke Keong Yong

**Affiliations:** ^1^Department of Human Anatomy, Faculty of Medicine and Health Sciences, Universiti Putra Malaysia, Serdang 43400, Selangor, Malaysia; ^2^Department of Anatomy, Faculty of Medicine, Universiti Kebangsaan Malaysia Medical Centre, Cheras, Kuala Lumpur 56000, Malaysia; ^3^Department of Biomedical Science, Faculty of Medicine and Health Sciences, Universiti Putra Malaysia, Serdang 43400, Selangor, Malaysia; ^4^Advanced Medical and Dental Institute, Universiti Sains Malaysia, Bertam 13200, Kepala Batas, Penang, Malaysia; ^5^Department of Pre-Clinical Sciences, Faculty of Medicine and Health Sciences, Universiti Tunku Abdul Rahman, Kajang 43000, Selangor, Malaysia; ^6^Department of Oral Biology and Basic Sciences, Faculty of Dentistry, Universiti Sains Islam Malaysia, Persiaran MPAJ, Jalan Pandan Indah 55100 Pandan Indah, Ampang, Malaysia

## Abstract

Vascular endothelial dysfunction is characterized by an imbalance of vasodilation and vasoconstriction, deficiency of nitric oxide (NO) bioavailability and elevated reactive oxygen species (ROS), and proinflammatory factors. This dysfunction is a key to the early pathological development of major cardiovascular diseases including hypertension, atherosclerosis, and diabetes. Therefore, modulation of the vascular endothelium is considered an important therapeutic strategy to maintain the health of the cardiovascular system. Epidemiological studies have shown that regular consumption of medicinal plants, fruits, and vegetables promotes vascular health, lowering the risk of cardiovascular diseases. This is mainly attributed to the phytochemical compounds contained in these resources. Various databases, including Google Scholar, MEDLINE, PubMed, and the Directory of Open Access Journals, were searched to identify studies demonstrating the vascular protective effects of phytochemical compounds. The literature had revealed abundant data on phytochemical compounds protecting and improving the vascular system. Of the numerous compounds reported, curcumin, resveratrol, cyanidin-3-glucoside, berberine, epigallocatechin-3-gallate, and quercetin are discussed in this review to provide recent information on their vascular protective mechanisms in vivo and in vitro. Phytochemical compounds are promising therapeutic agents for vascular dysfunction due to their antioxidative mechanisms. However, future human studies will be necessary to confirm the clinical effects of these vascular protective mechanisms.

## 1. Introduction

Epidemiological studies in the last few decades have demonstrated that the consumption of foods containing high levels of flavonoids such as curcumin, resveratrol, cyanidin-3-glucoside, berberine, epigallocatechin-3-gallate, and quercetin was associated with a lower risk of cardiovascular morbidity and mortality [1]. The beneficial effect of these phytocompounds in the cardiovascular system was partly due to direct effects towards the blood vessels and the endothelium. Evidence from cohort studies and pivotal research demonstrated the ability of phytocompounds to constantly modulate vasoprotective factors. These factors regulate the function of the endothelial barrier [2,3]. These compounds show promise in the management of cardiovascular disease.

Cardiovascular disease is a leading cause of mortality globally, with a projected increase in the coming years, hence placing a tremendous strain on the world's health resources [4]. Poor diet, smoking, obesity, and physical inactivity are among the well-known modifiable risk factors for cardiovascular disease, all of which are conditions associated with inflammation [5]. Inflammatory response plays a crucial role in endothelial cell activation/dysfunction and the pathogenesis of cardiovascular diseases [6]. Therefore, new therapeutic targets are sought to prevent cardiovascular diseases.

Although effective, medications are also associated with adverse effects and cost, as well as challenges with patient compliance [7]. On the other hand, herbal medicine with adequate clinical effectiveness and low toxicity has been used as an alternative to treat diseases [8,9]. Natural compounds such as flavonoids are becoming increasingly popular. They offer a variety of therapeutic benefits, including anti-inflammatory and antioxidizing properties, which are critical in the treatment of cardiovascular disorders [10]. In this paper, a few phytocompounds of interest are reviewed for their vascular endothelial cell protective effects against proinflammatory mediators. These compounds were isolated from natural products such as plants, vegetables, and fruits. In addition, the possible mechanisms by which these compounds can improve vascular endothelial cell integrity were elucidated.

## 2. The Functions of Vascular Endothelium under Physiological and Pathological Conditions

Endothelial cells of the vascular system line the entire circulatory system from the heart to the tiniest capillaries. The endothelial layer consists of 1 to 6 × 10^13^ cells, covers an area of approximately 1–7 m^2^, and weighs approximately 1 kg in an adult human [11]. The vascular system regulates blood fluidity and fibrosis, vascular tone, angiogenesis, vascular permeability, leukocyte adhesion, and platelet aggregation, all of which are important in maintaining the homeostasis of the cardiovascular system [12]. It is well established that a healthy vascular endothelium is an excellent predictor of cardiovascular health, whereas an aberrant vascular endothelium is invariably associated with a range of cardiovascular illnesses, including hypertension and atherosclerosis [13].

Additionally, endothelial cells form a semipermeable barrier that allows certain substances to pass through between the blood and the vascular wall. A variety of localized chemicals, including nitric oxide (NO), prostacyclin, endothelium-derived hyperpolarizing factor, and endothelin, are secreted by vascular endothelial cells to control the blood vessels [14]. These chemicals dilate blood vessels, promote anti-cell proliferation, are involved in antiagglutination, and reduce blood pressure under physiological conditions. These chemicals also alter endothelium homeostasis in pathological situations such as during hypercholesterolemia, vasoactive amine circulation, infection, and immunological conditions [15].

Endothelial cells also play an important function in blood flow regulation. This function is enhanced by the ability of quiescent endothelial cells to form an active antithrombotic surface that facilitates the flow of plasma and cellular components throughout the vasculature [16]. Endothelial cells generate a prothrombotic and antifibrinolytic milieu in response to perturbations, such as those that may occur during inflammation or high hydrodynamic shear stress [17]. Additionally, the production and absorption of vasoactive endothelial chemicals play a role in blood flow regulation. These chemicals work in a paracrine manner to constrict and dilate particular arterial beds in response to stimuli such as endotoxin [18].

The regulation of vascular tone is another essential function of the endothelium. Healthy endothelial cells help to maintain a balance between vasorelaxing and vasoconstricting factors in the blood. Endothelial cells release various vasoconstrictors such as angiotensin II, endothelin, thromboxane A2, and free radicals, while nitric oxide (NO) and prostacyclin (PGI2) are the most potent vasodilators to retain basal vascular tone. Endothelial cells synthesize these vasodilators in response to bradykinin, thrombin, adenosine triphosphate (ATP), adenosine diphosphate (ADP), and shear stress. Particularly, endothelial-derived NO stimulates the soluble guanylyl cyclase (sGC) pathway in smooth muscle cells which results in a reduction of contraction and vasodilation [14]. Hence, impaired bioavailability of NO is attributed to endothelial dysfunction, which initiates pathological events involving chronic inflammation such as atherosclerosis, hypertension, and diabetes [19].

Furthermore, the endothelium is also essential for coordinating leukocyte trafficking during vascular injuries. Lymphocytes, neutrophils, and monocytes do not adhere to the endothelium in physiological conditions due to the antiadhesive phenotype of endothelial cells. However, stimuli such as proinflammatory cytokines, shear stress, oxidized LDL (ox-LDL), and vasoconstrictors could shift the endothelial cell phenotype to be proadhesive to leukocytes, initiating the recruitment and the subsequent migration to the subendothelial space [20]. Here, the leukocytes develop into macrophages and then foam cells, assisting in the formation and progression of atherosclerotic plaques [21]. This shows that endothelial dysfunction and monocyte-endothelial interactions are critical for atherosclerosis' start and development.

**Figure 1:**
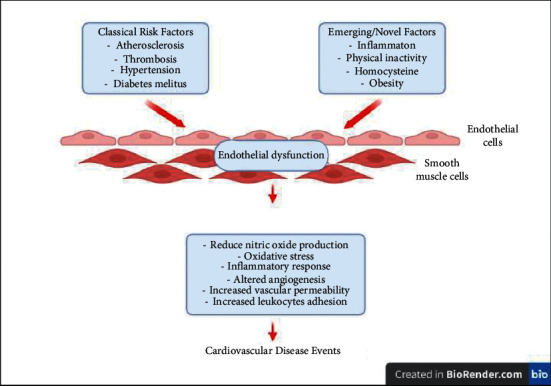
Mechanisms linked to endothelial dysfunction. Adapted from [26].

The development of vascular disease is dependent on the activation of the vascular endothelial cell. The activation increases the expression of proinflammatory mediators, chemokines, and growth factors that enhance ROS formation, inhibit eNOS expression, and decrease NO bioavailability [22], priming the blood vessels to be in a more prothrombotic and proinflammatory state [23,24]. Furthermore, the expression of cells adhesion molecules (CAM) such as E-selectin, vascular CAM-1 (VCAM-1), and intercellular adhesion molecules-1 (ICAM-1) is also upregulated, leading to leukocytes' recruitment, migration, and infiltration [25]. This mechanism occurs in many inflammatory diseases and induces endothelial cell inflammation and endothelial dysfunction [26]. The endothelial cell will have impaired vascular tone, endothelial-dependent vasodilatation, and redox imbalance as well as increased inflammatory reactions inside the wall of the blood vessel [27] (Figure 1). Proinflammatory mediators or cytokines that cause vascular endothelial cell activation and endothelial dysfunction are listed in Table 1. Identification of endothelial cell-derived inflammatory inducing factors and its underlying mechanisms may be beneficial in preventing the onset of cardiovascular diseases.

## 3. Vascular Protective Effects of Active Phytocompounds

### 3.1. Curcumin

Curcumin, a flavonoid compound derived from the roots of the plant *Curcuma longa,* is a major component of turmeric. Turmeric is commonly a spice and food-coloring agent. Curcumin's *ß*-diketone group, carbon-carbon double bonds, and phenyl rings with numerous hydroxyl groups and methoxy substituents make it a strong antioxidant [46]. Curcumin also has anti-inflammatory [47], antiviral, antibacterial, antifungal [48], anticancer, and antitumor effects [47,49], making it a promising phytochemical for treating cancer, diabetes, allergies, arthritis, Alzheimer's disease, and other chronic conditions [50,51].

Curcumin may also help protect endothelial cells from the negative vascular effects stimulated by TNF-*α* which modulates p38, signal transducer and activator of transcription-3 (STAT-3), nuclear factor kappa B (NF-*κ*B), and c-Jun N-terminal kinase (JNK) in endothelial cells [52]. Lee et al. [53] also report that curcumin significantly inhibited TNF-*α*-induced lectin-like oxidized LDL receptor-1 (LOX-1) expression and suppressed endothelial activation and dysfunction against TNF-*α*. Specifically, the authors demonstrated that curcumin treatment inhibited the formation of ROS, along with the degradation of I*κ*B*α* and the translocation of NF-B. Curcumin also simultaneously induced eNOS to ensure sufficient production and availability of NO for optimal endothelial function were not adversely affected [53]. Kim et al. [52] additionally found that curcumin reduced the production of ICAM-1 mRNA and its associated TNF-*α*-activated protein in human umbilical vein endothelial cells (HUVECs).

One of the prominent effects of curcumin on the endothelium is the inhibition of leukocyte adhesion. An *in vitro* study has shown that human umbilical vein endothelial cells (HUVECs) pretreated with 40 *μ*M curcumin for 1h completely inhibited endothelial cell adhesion to monocytes. The effect was induced by tumor necrosis factor-alpha (TNF-*α*) [52,54] and associated with the suppression of cell surface molecules. Particularly, ICAM-1, VCAM-1, E-selectin [54], monocyte chemoattractant protein (MCP)-1, and interleukin (IL)-8 were suppressed by curcumin at both the mRNA and protein level [52]. Leukocyte recruitment and adhesion are inflammatory hallmarks in the vasculature and probably the first stage in atherosclerotic plaque development [55]. Leukocytes adhered to the endothelial cells then differentiate into tissue macrophages, resulting in the development and stabilization of local inflammation. The matured macrophages, as well as their transition into foam cells, eventually form plaque. Hence, the vascular protective effect exhibited by curcumin is derived from the compound's ability to prevent leukocytes' recruitment, modulate lipoprotein composition, and attenuate oxidative stress through antioxidative processes.

Another factor that increases the risk of cardiovascular morbidity and mortality is environmental pollutants. [56]. For instance, the presence of high bisphenol A levels in urine and serum is associated with an increased risk and atherogenic alterations in the carotid artery, respectively [57]. Di-(2-ethylhexyl) phthalate (DEHP), on the other hand, is a popular plasticizer used in flexible polyvinyl chloride goods including soft-squeeze toys, food packaging, clothes, medical tubing, and blood storage bags. A growing body of epidemiological research indicates a favourable relationship between circulating levels of phthalate metabolites and cardiovascular diseases [58]. DEHP promoted atherosclerosis in mice model [59] and may enhance the expression of inflammatory mediators such as ICAM-1 and IL-8 in HUVECs via ERK and p38 MAPK signaling pathway. Increase in these markers increases the likelihood of allergic inflammatory reactions leading to hospitalization [60]. Interestingly, the same study reported that curcumin significantly inhibited ICAM-1 and IL-8 production stimulated by DEHP.

Extensive studies indicate that ambient exposure to environmental pollution is strongly associated with an increased risk of cardiovascular morbidity and mortality [56]. For instance, the presence of high urine bisphenol A levels relates to an increased risk of cardiovascular disease, and the elevated serum bisphenol A level is associated with atherogenic alterations in the carotid artery [57]. Di-(2-ethylhexyl) phthalate (DEHP), considered as one of the common environmental pollutants, is a popular plasticizer used in flexible polyvinyl chloride goods including soft-squeeze toys, food packaging, clothes, medical tubing, and blood storage bags. A growing body of epidemiological research indicates a favourable relationship between circulating levels of phthalate metabolites and cardiovascular diseases [58]. DEHP not only promoted atherosclerosis in mice model [59], study from in vitro also found that it may enhance inflammatory mediator expressions such as ICAM-1 and IL-8 in HUVECs via ERK and p38 MAPK signaling pathway, which increases the likelihood of allergic inflammatory reaction admissions [60]. Interestingly, Wang and Dong [60] reported that curcumin significantly inhibited ICAM-1 and IL-8 production which was stimulated by DEHP in the same study.

Pathogens have also been associated with disrupting the normal function of endothelial cells. Lipopolysaccharide (LPS) of Gram-negative bacteria cell walls is also a powerful monocyte/macrophages activator. The activation alters the production of important mediators including inflammatory cytokines and chemokines, leading to acute inflammation in many cell types including endothelial cells [61]. Furthermore, LPS-mediated release of high-mobility group box-1 (HMGB1) from macrophages and dendritic cells plays an important role in vascular inflammation [62,63]. A study revealed that curcumin inhibits LPS-mediated release of HMGB1 and HMGB1-mediated proinflammatory responses in human endothelial cells through downregulation of the cell surface expression of HMGB1 receptors TLR2 and TLR4 [64].

Curcumin injection has also been shown to reduce ICAM-1 expression using a mouse model of lung damage. The compound inhibited neutrophil sequestration by inhibiting the activation of endothelial-derived ICAM-1, which is normally observed in an endotoxemia mice model with induction of heme oxygenase (HO-1) [65]. Boola and colleagues [66] have shown that curcumin therapy for 6 weeks in male renovascular hypertensive rats inhibited the development of hypertension by reducing endothelial dysfunction, vascular remodeling, and oxidative stress. It has also been discovered that curcumin as a daily diet increases antioxidant activity, NO bioavailability, and decreases angiotensin-converting enzyme (ACE) and metalloproteinase-2 (MMP2) and MMP9 expression [66]. Curcumin supplementation at 300 mg/kg body weight reduced diabetic vascular inflammation in diabetes-induced endothelial dysfunction male Wistar rats [67]. The production of both ICAM-1 and the pro-oxidative NOX-2 enzymes was achieved by a reduction in ROS generation and leukocyte/endothelium interaction [67].

Moreover, in stroke-prone spontaneously hypertensive rats (SHRsp), treatment of curcumin at 100 mg/kg/day significantly delayed the onset of stroke and increased survival. These effects were a result of decreased ROS and improved endothelial-dependent relaxation of carotid arteries. In this study, both curcumin-mediated decrease of ROS and increase of NO production were blocked in the presence of UCP2 inhibitor genipin. This result was consistent with the *in vitro* study in which curcumin increased NO levels and decreased ROS levels in the HUVECs [68].

### 3.2. Resveratrol

Resveratrol (trans-3, 4, 5-trihydroxystilbene), a natural polyphenolic compound, is abundantly found in grape skin, peanuts, mulberries, and red wine. It is a phytoalexin that plants employ to protect themselves against fungus and other types of aggression. Epidemiological research connecting red wine to decreased mortality from cardiovascular diseases in the French population despite the high intake of dietary cholesterol and saturated fats was later associated with resveratrol [69]. Resveratrol has antioxidative, modulatory lipoprotein metabolism, antiplatelet aggregatory, anti-inflammatory, and antitumor properties [70–72]. Resveratrol also protected the cardiovascular system in a multidimensional way, including vasculature. The protective effect of resveratrol against vascular dysfunction has been demonstrated in *in vitro* and *in vivo* studies.

Human coronary artery endothelial cells treated with resveratrol significantly suppressed reactive oxygen species (ROS) production and NF-*κ*B activation while concurrently downregulated the ICAM-1, iNOS, IL-6, and TNF-*α* inflammatory markers against induction from TNF-*α* and cigarette smoke extract [73,74]. Resveratrol also protects vascular inflammation by targeting cyclooxygenease-1 (COX-1) and cyclooxygenase-2 (COX-2) that also reduces prostaglandin activity [71]. COX-1 is a constitutive isoform of COX regularly secreted in different sites including vasculature while COX-2 is an inducible isoform that releases prostaglandins, prostacyclines, and thromboxane, all of which cause inflammation [75]. In healthy blood vessels, endothelial and smooth muscle cells express COX-1, with COX-2 a distant second. However, in endothelial cells of both healthy and diseased blood vessels, COX-1 is a significant source of prostaglandins [76]. Overproduction of prostaglandins leads to the formation of prostaglandin *D*_2_ and *E*_2_, prostacyclin, and thromboxane *A*_2_ which eventually promote vascular inflammation and platelet aggregation [76]. Thus, the suppression of COX-1, COX-2, prostaglandin activity, and other inflammatory mediators by resveratrol protects vasculature from phlogistic agent-induced damage. Another study has also shown that resveratrol activates anti-inflammatory pathways, including sirtuin 1, [69] while *in vitro* studies demonstrated the compound's antioxidant potential.

Resveratrol reduces ROS generation by inhibiting nicotinamide adenine dinucleotide phosphate oxidases and scavenges hydroxyl, superoxide, metal-induced radicals, and hydrogen peroxide [77–80]. A study by Chen and team [81] demonstrated that high-glucose-induced oxidative stress and apoptosis in murine brain microvascular endothelial cell bEnd3 through the NF-*κ*B/NADPH oxidase/ROS pathway was significantly abrogated by resveratrol treatment. Resveratrol also attenuated oxidative injury in human vascular endothelial cells through the regulation of mtROS homeostasis, which in part was mediated through the activation of the SIRT3 signaling pathway [82]. Although resveratrol was capable to counter oxidative stress-induced vascular injury, the vascular protective effects are likely attributed to the indirect upregulation of the endogenous cellular antioxidant systems rather than its direct ROS scavenging activity. Additionally, resveratrol causes the increase of endogenous antioxidant enzymes in endothelial cells, including superoxide dismutases (SOD) [83], SOD1, glutathione peroxidase 1 (GPx1) and NADPH oxidase subunit (NO×4) [84], thioredoxin-1 (Trx-1) [85], heme oxygenase-1 [86], and NAD(P)H:quinone oxidoreductase (NQO1 and NQO2) [87].

Apart from abovementioned mechanism, resveratrol was also reported to protect the vascular system via the antiapoptotic effect. Specifically, resveratrol prevented circulating endothelial cells from oxLDL-induced apoptotic insults by downregulating Lox-1-mediated activation of the Bax-mitochondria-cytochrome c-caspase protease pathway [88], and increasing the expression of endothelial nitric oxide synthase (eNOS), which results in increased nitric oxide generation from endothelial cells [89]. Increased endothelial nitric oxide production can improve endothelial function and acutely reduce systolic blood pressure, which may be critical in preventing cardiovascular disease [90].

The vascular protective effect of resveratrol in animal models is also well documented. Male Wistar rats treated with 50 mg/L of resveratrol for 10 weeks significantly prevented high-fructose corn syrup-induced vascular dysfunction associated with a metabolic syndrome [91]. In addition to insulin resistance, hypertriglyceridemia, and hepatic steatosis, rats on a high-fructose diet also developed endothelial dysfunction [92]. The related mechanisms are decrease in nitric oxide synthase (NOS) activity and increase in ROS generation mediated by the vascular renin angiotensin system, which possibly results in lower NO bioavailability [92]. Resveratrol also significantly mitigated high-fat diet-induced vascular dysfunction in male C57BL/6 mice [93]. As obesity has a strong link with insulin resistance [94], insulin signal loss in endothelial cells promotes leukocyte-endothelial cell interactions, triggering inflammation associated with the development of vascular dysfunction [95]. The vascular protective effect of resveratrol in both metabolic disorders might be attributed to the increased expression of the eNOS and SIRT1 protein [91,93]. Moreover, resveratrol also promoted the integrity of epithelial cell lining, as well as suppressed leukocyte extravasation, which eventually prevented injury of the vascular [93].

### 3.3. Cyanidin-3-Glucoside (C3G)

Anthocyanins are polyphenols of the flavonoid family, a natural pigment responsible for the blue, purple, red, and orange colours widely present in fruits and vegetables. Anthocyanins are claimed to have potential health-promoting effects. In recent decades, the relatively high intake of anthocyanins is investigated for roles in health promotion and illness prevention. Cyanidin-3-glucoside (C3G), one of the major components of anthocyanins commonly found in black currant, red cabbages, red raspberry, blueberry, blackberry, and purple rice bran, contributes to the violet, blue, and red colours [96]. *In vitro* and *in vivo* studies demonstrated the antioxidant and anti-inflammatory properties of C3G [97,98], as well as providing protection against endothelial dysfunction, vascular failure, and myocardium damage [99,100]. It also has been shown to prevent obesity and hyperglycemia [101] and appear to help prevent cardiovascular diseases [102]. C3G and its phenolic metabolites also exhibited distinct biological properties and potentially beneficial effects in various human pathologies [103]. The vascular protective effect of C3G was mainly reported in cell culture models.

Sivasinprasasn et al. [104] and Pantan et al. [105] evaluated the effect of C3G on vascular endothelial cells (EA.hy926) and human aortic vascular smooth muscle cells (HASMCs) in an inflammation model against angiotensin II. Both studies reported that C3G pretreatment significantly suppressed NF-kB signaling pathway through downregulation of NF-kB p65, decreased the expression of inducible nitric oxide synthase (iNOS), enhanced nuclear erythroid-related factor 2 (Nrf2), increased superoxide dismutase (SOD), and increased heme oxygenase (HO-1) activities [104,105]. Pantan and team further investigated the possible vascular protective effect of C3G on angiotensin II-induced human aortic smooth muscle cells (HASMCs) inflammation. C3G significantly suppressed angiotensin II-induced HASMCs proliferation and migration through MAPK and PI3K/Akt pathways [106]. Furthermore, a combination of low-dose statins and C3G possessed a synergistic effect on inhibiting angiotensin II-induced inflammation in vascular smooth muscle cells [105,106]. A combination of atorvastatin and C3G successfully decreased the expression of ICAM-1 and VCAM-1, nitric oxide (NO) production, and NOX1 while concurrently increased NAD(P)H:quinone oxidoreductase (NQO-1) and glutamate-cysteine ligase catalytic subunit (*γ*-GCLC) [106].

Inflammation and oxidative stress-induced vascular endothelial dysfunction and phenotypic switch of vascular smooth muscle cells are the main factors contributing to the early stage of atherosclerosis [107,108]. Atherosclerosis is established to be a vascular inflammatory disease. Lesion areas in the vascular smooth muscle cells exhibited increased permeability, leukocytes' migration, foam cells' formation, and release of proinflammatory cytokines, which trigger a deleterious event such as myocardial infarction [109]. Vascular smooth muscle cells' proliferation and migration are also triggered by angiotensin II which promotes the formation of reactive oxygen species (ROS) by activating NADH/NADPH and subsequent activation of the NF-kB signaling pathway [110,111], leading to vascular dysfunction. Furthermore, angiotensin II promoted cellular hypertrophy, induced the release of various growth factors, altered the extracellular environment by modifying the matrix composition, and stimulated apoptosis in vascular smooth muscle cells [112]. Thus, the ability of C3G to suppress angiotensin II potentiates the compound against atherosclerosis.

Studies have also found that C3G modulates anti-inflammation in human umbilical vein endothelial cells' injury caused by TNF-*α* and lipopolysaccharide (LPS) both *in vitro* and *in vivo* [113,114]. Speciale and team [113] demonstrated that pretreatment of C3G at 20–40 *μ*M significantly suppressed TNF-*α*-induced HUVECs injury in a dose-dependent manner. The protective effects were associated with suppression of adhesion molecules expression, leukocyte adhesion, and antioxidative stress via the NF-kB signaling pathway. C3G also prevented oxidative stress, enhanced the endogenous antioxidant system, and activated the NrF2/ARE signaling pathway [115]. In addition, C3G inhibited vascular smooth muscle cells' proliferation through the repression of NOX activator 1, which is associated with the involvement of the STAT3 signaling induced by TNF-*α* [116]. Ma et al. [114] demonstrated that C3G positively reversed the effects of LPS by inhibiting NF-*κ*B and MAPK pathways on HUVECs and in mice models. The vascular protective effects of C3G were also documented against different types of vascular damaging factors, including peroxynitrite [99] and palmitate [117,118]. These studies demonstrate that C3G exhibited vascular protective effects through antioxidant activities by directly or indirectly blocking oxidative stress and enhancing the endogenous antioxidant system.

More recent study by Wang and his team [119] demonstrated the vascular protective effect of C3G in high-fat diet plus balloon catheter injured rabbit model. Based on the data obtained, C3G improved the function of endothelial cells and suppressed blood lipids. In this experiment, C3G may have downregulated miR-204-5p, which led to the upregulation of sirtuin 1 (SIRT1) and the restoration of endothelial cell functions [119]. In addition to this, mice supplemented with C3G significantly reversed hypercholesterolemia-induced endothelial dysfunction in both prevention and intervention studies [120]. Data from the animal studies fully supported the hypothesis from previous *in vitro* studies. The data suggested that the C3G reduces cholesterol and 7-oxysterol levels through the ATP binding cassette subfamily G member 1 (ABCG1) pathway, lowering superoxide generation and increasing eNOS activity and NO bioavailability, therefore alleviating hypercholesterolemia-induced endothelial dysfunction and atherosclerosis [120].

### 3.4. Berberine

Berberine (BBR), or 5,6-dihydro-9,10-dimethoxybenzo[g]-1,3-benzodioxolo [5,6-á] quinolizinium, is an isoquinoline alkaloid mainly found in rhizomes, roots, and stem bulk of plants such as the Berberidaceae and Ranunculaceae families, and Chinese herb Huanglian. Based on the chemical formula of BBR, C_20_H_18_NO_4_ is a protozoan morphinane alkaloid [121]. It has long been used in the treatment of gastrointestinal infections and diarrhoea but more recently is reported to be used in treating cardiovascular complications [122]. BBR has been extensively studied on its pharmacological activity *in vivo* and *in vitro* settings, in which it exhibited anticancer [123], anti-inflammatory [124], antileishmanial [125], anti-human immunodeficiency virus [126], and neuroprotective agent [127]. Cardiovascular protective effects were revealed based on evidence that BBR suppressed proliferation and migration of vascular smooth muscle cells [128], blocked macrophages-derived foam cell formation [129], anti-inflammatory functions, inactivated inflammasome [130], inhibited platelet activation [131], and normalized vascular endothelial function [132].

BBR treatment also intervened oxidized low-density lipoprotein (oxLDL)-stimulated endothelial dysfunction. oxLDL is a proatherogenic lipoprotein that leads to excessive or abnormal proliferation of vascular endothelial cells eventually leading to atherosclerotic plaque formation [133]. Hsieh and his team [132] documented that BBR perturbed ROS production triggered by oxLDL on HUVECs. Moreover, BBR protected the HUVECs by reducing cytotoxicity and apoptosis induced by oxLDL. This effect was attributed to the regulation of mitochondrial transmembrane permeability, reducing proapoptotic protein while increasing antiapoptotic proteins [132]. Xu et al. [134] further reported that the antiproliferative and anti-inflammatory activities of BBR were exerted by lowering the expression of NF-кB, lectin-like oxidized low-density lipoprotein (LDL) receptor-1 (LOX-1), proliferating cell nuclear antigen (PCNA), and inhibiting the phosphorylation of the PI3K/Akt, extracellular signal-regulated protein kinase (ERK1/2), and p38 mitogen-activated protein kinases (MAPKs) on HUVECs, as stimulated by oxLDL.

Similar to other proinflammatory cytokines, oxLDL is capable of activating vascular endothelial cells and subsequently the expression of a series of adhesion molecules, which leads to leukocyte migration and adhesion [135]. HUVECs were also reported to be protected by BBR when challenged with oxLDL through the inhibition of VCAM-1 and ICAM-1 expression and adhesion of monocytes [136]. Interaction between oxLDL with its scavenger receptor, the lectin-like oxLDL receptor 1 (LOX1), was shown to stimulate endothelial expression and secretion of proatherogenic enzymes as well as the production of NOX (nicotinamide adenine dinucleotide oxidase)-derived ROS, thereby decreasing local NO level [137]. Interestingly, BBR suppressed LOX1 expression in the HUVECs through counterreaction with NOX2-derived ROS, MAPK/Erk1/2, and NF-*κ*B activation [138]. More surprisingly, BBR exhibited higher activity compared to that of the reference drug, lovastatin [138].

Studies have also revealed that BBR effectively inhibited inflammatory effects caused by TNF-*α* in cultured human aortic endothelial cells (HAECs) and HUVECs [138,139]. BBR could prevent TNF-*α*-induced LOX1 expression and oxidative stress, possibly through the NOX, MAPK/Erk1/2, and NF-*κ*B signaling cascade [138]. BBR was shown to abrogate the vascular endothelial damaging effect of TNF-*α* by attenuating the production of ICAM-1 and MCP-1 through suppression of NF-*κ*B following AMPK activation in the HAECs [139]. BBR also significantly reverted vascular endothelial cell injury caused by other damaging factors. For instance, BBR suppressed leukocyte adhesion and expression of adhesion molecules in both *in vivo* and *in vitro* endothelial cell models. In these models, the damage was induced by LPS via blocking the NF-*κ*B signaling pathway [140].

BBR protected vascular endothelial cells against angiotensin II by reducing intracellular ROS production, MCP-1, and monocyte adhesion [141]. In addition, BBR was also capable of enhancing NO production in vascular endothelial cells via upregulation of eNOS expression and its activity, in which the underlying mechanism might involve upregulation of AMP-activated protein kinase (AMPK) and downregulation of NOX4 activities [142]. Furthermore, Cheng and his team [143] revealed the vascular endothelial protective effect of BBR via a clinical trial and *in vitro* model. According to the data, BBR significantly reduced serum malondialdehyde (MDA) level and circulating endothelial microparticles in human sample, which were correlated with improvement of flow-mediated vasodilation [143]. Moreover, CD31+/CD42 circulating endothelial microparticles have emerged as a sensitive marker for endothelial damage in response to a variety of stimuli, including inflammation, partially via increasing oxidative stress in endothelial cells [144,145]. In a cell culture setting, BBR also significantly reversed the vascular endothelial injury caused by endothelial microparticles, by altering the oxidative stress level [143].

### 3.5. Epigallocatechin-3-Gallate

Green tea (Camellia sinensis), a beverage widely diffused and consumed in the Asian population, has long been known for its extensive health benefits, which are mainly attributed to its polyphenol content [146]. In fresh leaf in dry weight, approximately 30% is made up of polyphenol contents, particularly flavanols and flavonols [147]. Catechins are the major flavonoids found in green tea [147], and among the catechins, epigallocatechin-3-gallate (EGCG) is mainly present in green tea. Extensive research has revealed that EGCG exhibited antioxidative stress [148], anti-inflammation [149], anticancer [150,151], antimicrobial [152], neuroprotective [153], hepatoprotective [154], renal protective [155], and cardiovascular protective effects [156].

The phenol rings in EGCG's structure trap electrons and scavengers' free radicals [157], conferring the capability to inhibit the formation of ROS, and consequently the harming effects of oxidative stress [158]. EGCG had also effectively suppressed platelet activation induced by collagen or thrombin [159] and improved the function of mitochondria [160]. However, studies have shown that EGCG at high concentrations can cause self-oxidization and function as a prooxidant [161,162]. This occurs via the production of hydroxyl radicals, hydrogen peroxide, and quinonoid intermediates which can cause cytotoxicity [163]. Therefore, more evidence is required to evaluate the relationship between dose and antioxidant signaling pathways in cellular activity.

The vascular protective effect of EGCG was observed in a TNF-*α*-induced inflammation cell culture study with HUVECs. EGCG significantly reduced MCP-1 and monocyte adhesion in a dose-dependent manner via inhibition of NF-*κ*B signaling cascade against [164]. Furthermore, Yang and team demonstrated that EGCG suppressed the inflammatory response of vascular endothelial cells by increasing the cytosolic calcium (Ca^2+^) triggered by TNF-*α* [165]. Elevation of cytosolic Ca^2+^ causes the activation of disintegrin and cleavage of metalloprotease 10 (ADAM10), followed by ectodomain shedding of tumor necrosis factor receptor 1 (TNFR1) [165]. By decreasing the number of receptors on the endothelial cell surface, the shedding of TNFR1 ectodomain reduces the cell's sensitivity towards TNF-*α*. Furthermore, the released ectodomain part of TNFR1 may bind to TNF-*α* in the extracellular space and negate its action [166]. A study revealed that EGCG potentially suppressed the inflammatory response of vascular endothelial cells against TNF-*α* via ADAM-mediated ectodomain shedding of TNFR1 [167]. Moreover, treatment of EGCG on human coronary artery endothelial cells caused the downregulation of multiple cytokines, chemokines, and transcription factor activity, including NF-*κ*B-p65 DNA-binding activity, MCP1, IL-6, and IL-8 stimulated by TNF-*α* [168]. Moreover, EGCG also significantly reversed endothelial hyperpermeability and monocytes stimulated by TNF-*α* [168].

The treatment of HUVECs with EGCG caused a reduction of oxidative stress factors including ROS, nitrite, and MDA, while concurrently enhancing the SOD and GSH antioxidant enzymes [169]. EGCG significantly prevented HUVECs apoptosis and expression of adhesion molecules by inhibiting NF-*κ*B activation [169]. A study has additionally shown that EGCG abrogated MC-LR-induced apoptosis of HUVECs through activation of NRF2/HO-1 signaling [170]. Microcystin-LR (MC-LR) is a toxin released from aquatic cyanobacteria and causes a variety of adverse effects on different cell types, tissues, and organs, including liver [171], kidney [172], cardiovascular, lung, intestine, and spleen [173]. MC-LR has also been shown to cause apoptosis in HUVECs by inducing oxidative stress via NF-*κ*B activation [174]. EGCG counteracted NADPH oxidase, particularly p47^phox^, in which this angiotensin II-mediated oxidase was responsible to initiate and develop inflammatory vascular diseases and inhibited the expression of iNOS induced by angiotensin II in HUVECs [175,176]. However, EGCG failed to suppress ROS in angiotensin II-stimulated HUVECs [175,176]. EGCG has also been reported to effectively protect vascular endothelial function from high-glucose stress-induced injury [177].

In addition to vascular protective effects in the *in vitro* models, EGCG also ameliorated endothelial dysfunction in animal models. For instance, Potenza and team evaluated the impact of EGCG administration on the ability of spontaneously hypertensive rats (model of metabolic syndrome with hypertension, insulin resistance, and overweight) to enhance both their cardiovascular and metabolic performances at the same time [178]. Here, EGCG exhibited a vasodilator effect in mesenteric vascular beds *ex vivo*, in both acute and chronic studies. The study suggested that the vasodilator effect was in part attributed to the stimulation of nitric oxide production from the endothelium through the PI 3-kinase-dependent pathway [178]. Furthermore, obese mice fed with EGCG at 50 mg/kg/day for 10 weeks showed improved insulin sensitivity, glucose tolerance, and endothelial function [177]. As obesity is highly associated with various metabolic and cardiovascular disorders, including insulin resistance, diabetes, and atherosclerosis [179], the pathogenesis of diabetes and its associated cardiovascular consequences is heavily influenced by reciprocal interactions between insulin resistance and endothelial dysfunction [180]. Thus, altering high-fed-diet-induced inflammatory response in obese mice, especially the amelioration of insulin resistance by EGCG, might be beneficial for endothelial function.

### 3.6. Quercetin

Quercetin (3,3′,4′,5,7 pentahydroxy flavone) is a natural flavonoid widely and abundantly found in almost all plant food such as tea, onion, lettuce, broccoli, beans, fruits, and buckwheat. It is also one of the effective components of ginkgo leaves, mulberry parasitic, sandalwood, and other Chinese herbs [181]. Accumulating evidence suggests that quercetin exhibits antioxidant, anti-inflammatory, and antimicrobial activities [182,183]. Moreover, recent studies have found that quercetin can restrain the proliferation and metastasis of multiple cancer cell types such as breast cancer and colon cancer [181]. The presence of five hydroxyl groups in the structure of quercetin confers a significant level of antioxidant activity. Furthermore, the pyrocatechol type of the benzene ring makes it a strong scavenger of free radicals [184]. Quercetin is one of the most often consumed members of the flavonoid family, accounting for around 65–75% of our daily intake of flavonoids [185]. Quercetin may be found in nature in the form of ramifications that attach to glucose and rutinose [186]. Once consumed, quercetin can be swiftly degraded by the glucosidase enzyme in the digestive system, making it simpler for absorption by the mucosa of the large intestine and subsequently transported to the entire body through the portal circulation [187]. Quercetin is nontoxic and nonlethal to animals, even at large dosages (4000 mg/day), making it a safe supplement for human use [188].

Quercetin protects vascular endothelial injury through multiple approaches, for instance through its antioxidative effects. Treatment of quercetin protected the vascular endothelial cells by scavenging ROS, increasing the level of SOD and HO-1, while concurrently decreasing MDA and XO-1 levels in TNF-*α* and homocysteine-induced vascular endothelial cells [189,190]. Moreover, quercetin significantly opposed tunicamycin-induced HUVECs injury by reducing endoplasmic reticulum stress, possibly through the poly (ADP-ribose) polymerase (PARP) signaling [191].

Quercetin also demonstrated encouraging benefits for vascular endothelial cells' dysfunction and endothelial cell activation triggered by diabetes-related high-glucose levels in the circulation. Endothelial cells exposed to a hyperglycemic environment produce less nitric oxide but had increased cell adhesion molecules and inflammatory gene expressions caused by activation of NF-кB signaling [192]. In addition, the hexosamine biosynthesis pathway becomes overactive in diabetic individuals, leading to elevated levels of glucosamine in the blood circulation [193]. Increased glucosamine will cause endoplasmic reticulum stress, which may lead to exacerbation of endothelial cell damage. The damage eventually leads to increased inflammation and lipid metabolic abnormalities, which eventually accelerate the development of atherosclerosis [194]. A study done by Ozyel and the team [192] reported that quercetin was capable of regulating the balance of HUVECs metabolites towards a less inflamed phenotype when challenged with high-glucose stimuli. Furthermore, findings from Cai et al. [195] revealed that quercetin's protective benefits against high-glucosamine-induced HUVECs damage may be mediated through the ER/CHOP and ER/JNK pathways. PERK may also be a crucial component of the molecular mechanism responsible for these protective effects. More recently, quercetin was shown to inhibit vascular endothelial inflammation in diabetic vasculature, via suppression of myeloperoxidase/high-glucose-dependent hypochlorous acid formation. The acid formation plays an important role in diabetic vascular complications [196].

Quercetin also showed promising data in animal models. Male Sprague-Dawley rats orally treated with quercetin at doses of 25 or 50 mg/kg/day for 6 days significantly ameliorated vascular dysfunction challenged by phenylhydrazine (PHZ) [197]. PHZ is widely used for inducing haemolytic disorders and investigating anemic mechanisms [198]. It is a strong oxidant that exhibits a number of toxic effects, and the free radicals generated from PHZ might directly impact vascular tissues via oxidative stress-induced inflammation and reduction in NO bioactivity, eventually resulting in vascular dysfunction [197]. It is widely known that quercetin possesses a very strong antioxidant activity, capable of scavenging free radicals generated from PHZ and thus decreasing oxidative stress. In addition, quercetin restored arterial blood pressure and vascular responsiveness to endothelium-dependent vasodilators and vasoconstrictors, which ultimately prevent the vascular from PHZ damage [197]. Furthermore, oral administration of quercetin at 50 or 100 mg/kg either prior to or after exposure to LPS protected vascular function as measured by blood pressure, heart rate, and vascular responsiveness, which were all recovered to near-normal levels [199]. These effects were highly associated with upregulation of eNOS expression, a decrease of oxidative stress, and maintenance of the blood GSH redox ratio [199]. Quercetin also reduced inflammatory cardiovascular risk factors, such as serum amyloid A (SAA), C-reactive protein, and fibrinogen levels, when administered to mice fed with an atherogenic 1% cholesterol-containing diet [200]. Quercetin also significantly decreased histological atherosclerotic lesion growth. However, no effect was seen on monocyte adherence to the endothelium and on the lesion macrophage content [200]. The vascular protective effect of quercetin in animal models is mostly similar to other antioxidant compounds, as previously described through antioxidant activities.

## 4. Conclusion and Future Prospective

Natural herbs are rich sources of potential therapeutic candidates for various diseases including cardiovascular diseases, neurodegenerative disorders, and metabolic dysregulation. Repurposing the existing medications for alternative applications is an important way to discover new therapies with known safety profiles. For cardiovascular diseases, the pivotal components of pathogenesis are ROS production and inflammation, which are targeted by many phytocompounds and existing medications. Although extensive studies have been carried out to determine the vascular protective effects of active phytocompounds reviewed in this paper, there are ongoing developments and research studies on other human diseases. Active phytocompounds isolated from natural resources often face challenges in bioavailability and stability. Thus, improving extraction and formulation techniques to maintain biological activities is crucial. Furthermore, biosafety, long-term bioactivity, degrading properties, interactions with immune cells, the ability to sustainably circulate in humans, and excretion must be thoroughly evaluated before consumption. In addition, further research is needed to minimize the cost of industrial-scale manufacturing, develop better methods for synthesis or extract, and discover the optimal route of administration. New phytochemical agents to treat cardiovascular diseases particularly vascular endothelial cells are expected to surface with the progress of research.

## Figures and Tables

**Table 1:** Summary of the type of endothelial cell damaging factors with its effects on respective vascular endothelial cells.

Source	Type of endothelial cell	Effect/mechanism	Ref.
Tumor necrosis factor-alpha (TNF-*α*)	Human umbilical vein endothelial cells (HUVECs)	(i) Induction of oxidative stress, inflammation, and apoptosis (ii) Induction of inflammation and monocytes adhesion	[28,29]
Glycated low-density lipoprotein (glyLDL)	Porcine aortic EC (PAEC)	(i) Induction of oxidative stress and apoptosis	[30]
Bradykinin	HUVECs	(i) Induction of endothelial hyperpermeability (ii) Stimulation of angiogenesis via increased endothelial permeability and remodeling	[31,32]
Histamine	HUVEC Human dermal microvascular endothelial cells (HDMEC)	(i) Increased endothelial permeability through PLC-NO-cGMP signaling cascade (ii) Induction of endothelial dysfunction by activating Ca2+-mediated RhoA and adherens junctions' tension	[33,34]
*α*-Thrombin	HUVECs	(i) Increased endothelial macromolecular permeability	[35]
IFN*γ*	HUVEC	(i) Induction of endothelial hyperpermeability via activation of p38 MAP kinase and actin cytoskeleton alteration	[36]
IL-1*α*	Brain microvascular endothelial cells (BECs)	Activation and induction of angiogenic markers in endothelial cells	[37]
IL-1*β*	Human glomerular endothelial cell (HRGEC)	Induction of vascular hyperpermeability and upregulation of vascular endothelial-cadherin	[38]
IL-4	Human coronary artery endothelial cells (HCAEC) and human pulmonary artery endothelial cells (HPAEC)	Induction of vascular hyperpermeability through Wnt5A signaling	[39]
Lipopolysaccharide (LPS)	HUVECs HUVECs and human lung microvascular endothelial cells (HMVEC-L)	(i) Induction of apoptosis, injury, JNK phosphorylation, decreased MCL-1 expression and SOD activity, and increased proinflammatory cytokine production. (ii) Activation of endothelial cells' inflammatory responses	[40,41]
Thrombin	Primary human dermal microvascular endothelial cells (HDMECs) Human pulmonary microvascular endothelial cells (HPMVECs)	(i) Induction of microvessel leakage (ii) Induction of vascular hyperpermeability via dysregulation of vascular endothelial (VE-)cadherin and alteration of small rho GTPases	[42,43]
Angiotensin II (Ang II)	HUVECs	Induced vascular endothelial cells' injury and oxidative stress	[44]
Glucose	Rat aortic endothelial cells (RAOECs)	Induces cyclin D2 upregulation and miR‐98 downregulation	[45]

## Data Availability

The data sets supporting the conclusions of this study are included within the manuscript.
